# Early antibiotic treatment for gradual ventilator-associated pneumonia: yes or no?

**DOI:** 10.1186/s13054-016-1461-8

**Published:** 2016-10-25

**Authors:** Xuandong Jiang, Yanfei Shen

**Affiliations:** Intensive Care Unit, Dongyang People’s Hospital, No. 60 Wuning West Road, Jinhua City, 322100 Zhejiang People’s Republic of China

We read with interest the observational study by Paula Ramirez et al. [1] discovering the appropriate starting time of antibiotic treatment in patients with ventilator-associated pneumonia (VAP).

In that study, 440 patients receiving mechanical ventilation were screened, but only patients with VAP were studied, including 43 with gradual VAP and 28 with non-gradual VAP, and they suggested that early antibiotic treatment should be applied in gradual VAP patients.

However, in clinical practice, not all patients with gradual VAP symptoms would finally progress to VAP. According to a retrospective study [2], less than 10 % of the patients with nosocomial tracheobronchitis (NTB), with similar criteria to gradual VAP, would developed subsequent VAP (Tables 3 and 4 in [2]). As it is difficult to discriminate these patients until VAP has developed, it is crucial for clinicians to know whether earlier antibiotic treatment would benefit those patients with NTB but without subsequent VAP. Besides, Nseir et al. [2] reported that antibiotic use could not decrease the VAP incidence in patients with NTB. Thus, whether early antibiotic treatment will improve clinical outcome (such as VAP rate and mortality) or will lead to deterioration of antibiotics abuse based on the low proportion who developed VAP in NTB patients remains unclear?

The other question is that, in this study, unadjusted comparisons were made between two groups. The primary result was that, compared to the VAP group, antibiotic treatment in the gradual VAP group led to a higher rate of early clinical response (*p* = 0.009). No significant difference was found in hospital length of stay or mortality. These results were similar to the former study [2]. Compared to patients with gradual VAP, non-gradual VAP may indicate stronger inflammation which we thought to be a really important confounding factor of clinical response time. Caution should be raised when we interpret this outcome.

## Abbreviations

NTB: Nosocomial tracheobronchitis
VAP: Ventilator-associated pneumonia
