# Supplementary material for: Early antibiotic treatment for gradual ventilator-associated pneumonia: yes or no?
Source: Crit Care. 2016 Oct 25;20:340. doi: 10.1186/s13054-016-1461-8 (PMC5078934; doi:10.1186/s13054-016-1461-8)
Supplement: Related texts

# Reply: Authors’ response

**Authors:** Paula Ramírez, Mónica Gordón

As Dr Jiang and his colleagues expressed in their letter, there are still many unknowns regarding infectious complications in the mechanically ventilated patient.

We have focused on the study of the VAP maturation process suffered by a significant number of ventilated patients [1]. Our main conclusion is that trying to draw a diagnostic line on most of the late-onset pneumonias is only a response to an attempt to oversimplify the situation. We noted, like other authors [3], that the introduction of an appropriate treatment even in the absence of a complete diagnosis of VAP achieved an improved prognosis in our patients. Unfortunately, our study was not able to identify any tool that is able to properly guide the initiation of antibiotic treatment.

We believe that, given recent results obtained in the study of the so-called ventilator-associated tracheobronchitis as well as our own conclusions, it is necessary to shift the methodology of studies on VAP. The next clinical trials should be designed according to a dynamic scheme to evaluate the efficacy and safety of different strategies to identify the right time to start and end antibiotic treatment. Moreover, the chosen strategy should also be evaluated in terms of ecological sustainability (excessive selection of multi-resistant bacterial strains) [4].

In conclusion, far from solving all the unknowns mentioned by Jiang et al., our ultimate aim is to encourage experts in the field to change their approach to VAP in a more consistent way to acheiev clinical reality.
